# Consumers’ Fears Regarding Food Availability and Purchasing Behaviors during the COVID-19 Pandemic: The Importance of Trust and Perceived Stress

**DOI:** 10.3390/nu12092852

**Published:** 2020-09-17

**Authors:** Marzena Jeżewska-Zychowicz, Marta Plichta, Maria Królak

**Affiliations:** Department of Food Market and Consumer Research, Institute of Human Nutrition Sciences, Warsaw University of Life Sciences (SGGW-WULS), Nowoursynowska 159C, 02-776 Warsaw, Poland; marta_plichta@sggw.edu.pl (M.P.); maria.krolak@gmail.com (M.K.)

**Keywords:** COVID-19 pandemic, food purchase, consumer, trust, fears, perceived stress

## Abstract

The present study aimed to investigate whether trust in circulating information and perceived stress are predictors of consumers’ fear of limited access to food as well as predictors of food purchase behaviors during the COVID-19 pandemic. The computer-assisted web interviewing (CAWI) technique was used to collect data from 1033 Polish adults in March 2020. Logistic regression was used to estimate the likelihood of fear of limited access to food and the likelihood of purchase of larger amounts of food than usual. The likelihood of experiencing fear of limited access to food increased by 16% with higher perceived stress, by 50% with higher trust in “Mass media and friends”, and by 219% with perceived changes in food availability in the previous month. Trust in “Polish government institutions” decreased the chance of experiencing such fears by 22%. The likelihood of purchasing larger quantities of food than usual increased by 9% with higher perceived stress, by 46% with higher trust in “Mass media and friends”, by 81% with perceived changes in food availability in the last month, and by 130% with fears of limited access to food as the pandemic spreads. Government institutions may have difficulty in disseminating pandemic-related recommendations through media, not only due to relatively low trust people have in media organizations but also due to the increasing likelihood of the occurrence of both fears regarding food availability and panic-stricken food-buying behaviors with increase in trust in this source of information. Therefore, it is necessary to develop interventions that will reduce perceived stress and improve the trust in information from reputable sources.

## 1. Introduction

The appearance of SARS-CoV-2 in late December 2019 in Wuhan triggered a new, rapidly evolving situation, with the spread of the virus outside China. In March 2020, the vast spread of the COVID-19 disease in the world qualified it as a global pandemic [1]. In most countries, legal regulations were introduced to slow down the virus spread [2], including the order of social isolation (lockdown), use of masks, travel restrictions, etc. [3]. In Poland, an epidemic threat was announced on 12 March 2020, followed by the announcement of the state of pandemic on 20 March 2020. Along with the changes in the legal regulations, significant changes occurred in the food market. From 11 March 2020 onward, the turnover in grocery stores rocketed, queues increased, and shortages of goods began to appear. The Ministry of Development attempted to calm concerns regarding food shortages by providing information that denied rumors about the closure of retail outlets or the placement of sanitary cordons around cities [4]. The use of face masks was made mandatory only on 16 April 2020. Generally, restrictions in force in Poland and other countries inevitably caused public concerns, which were further magnified by mass media and social media coverage, misinformation, and pseudoscience [5,6,7,8].

For the majority of Poles, grocery stores became one of the few public places visited during the lockdown. Thus, shopping for food could increase the risk of contracting SARS-CoV-2 infection due to sharing of indoor space [9,10,11]. Previous studies have revealed that consumers feared contracting SARS-CoV-2 infection during grocery shopping [12,13]. In addition to this perceived fear, strong concerns about the availability of food favored increased purchasing activity, which led to short-term shortages of food and cleaning products, the so-called empty shelves, which Poles have already experienced before during the 1970s and the 1980s [14]. As in other countries, to calm the situation, government institutions persuaded citizens that supplies were maintained at a level that ensured access to food and other products [15,16]. Mass media were active in broadcasting information regarding pandemic and food supplies [17]. Although real shortages in access to food appeared briefly, mental and social functioning was significantly deteriorated due to stress arising from many unknowns, also related to access to food, social isolation, unusual work mode, and information flood [15,18,19].

During the COVID-19 pandemic, many people were forced to quarantine themselves at home, resulting in a high prevalence of psychological distress [20]. This stress was intensified by mass media that informed widely about the rapid increase in morbidity and mortality and the limited availability of diagnostic tests [21]. Thus, exploring consumers’ fears, perceived stress, and uncertainties about some aspects of food situation in the COVID-19 pandemic is desirable for many reasons. Changes in people’s food purchase patterns and food consumption due to social isolation directives as well as uncertainty about future may increase their fears uncontrollably and may affect eating patterns in various ways [13,22]. Changes in purchase patterns, e.g., bulk buying of foods, may lead to consuming food that is no longer safe, while stockpiling may lead to certain foods being available in stores in a very limited supply. Moreover, not only binge-eating but also consumption of palatable foods, snacking, and alcohol consumption may appear [23].

In a situation of uncertainty, appropriate communication about the situation concerning the COVID-19 pandemic becomes crucial for citizens [24]; this is because the public has limited knowledge about the infection and the modes of its spread [25]. Simultaneously, the loss of trust in information provided by government agencies as well as by doctors or nutritionists on TV or social media is observed [26]. Thus, enhancing trust in government and health authorities is required for a greater focus [27]. Trust is understood as a level of subjective probability with which the public (trustor) assesses that the other agent (trustee) will perform a particular action and the context that affects trustor’s action [28] is critical in situations where perceived risk and/or lack of knowledge occur [29]. According to Giddens [30], trust leads to consciously or subconsciously making a “leap of faith,” i.e., acknowledging that the expertise required to address the lack is held by another individual or system. Receiving information can affect the public’s knowledge about perceived risk, thereby influencing their decisions to adopt protective measures [31,32]. However, public response to communication efforts may be shaped less by explanations of uncertainty than by trust in the parties involved [25,33]. It is, therefore, important to understand how the public perceives the situation and to what degree they trust different agents that inform them about uncertainty resulting from the COVID-19 pandemic [31,34]. The Trust and Confidence Model suggests that people with higher levels of trust or confidence in institutions are more likely to accept recommended measures than those with lower trust or confidence levels [35,36]. Studies examining the H5N1 influenza indicated that trust not only in health agencies but also in government and media positively influenced people’s willingness to adopt precautionary behavior during a pandemic [37,38,39]; however, the abundance of information or conflicting messages can decrease public trust [36].

Learning about food purchasing behavior is vital not only to understand how the behavior of consumers changes under stressful conditions but also to provide useful guidance for some management efforts. However, public trust in available information is crucial in order to succeed in the efforts undertaken. To address the abovementioned issues, the present study aimed to investigate whether trust in information and perceived stress are predictors of consumers’ fears regarding limited access to food as the pandemic spreads and predictors of food purchase behaviors during the COVID-19 pandemic. We hypothesized that higher trust in the source of information and lower levels of perceived stress protect a person from experiencing fear regarding unavailability of food as well as from buying large amounts of food.

## 2. Materials and Methods

### 2.1. Data Collection

Data were collected between the 19th and 24th of March 2020 through a cross-sectional quantitative survey. In accordance with the study design, recruitment and data collection were conducted by a professional market research agency in accordance with the European Society for Opinion and Marketing Research (ESOMAR) code of conduct using the Computer Assisted Web Interview (CAWI) technique. The study sample was recruited from an e-panel of ARC Market and Opinion Agency that included around 60,000 registered individuals. The recruitment criterion was age; the respondents were between 18 and 65 years of age. Quota selection regarding gender, age, place of residence, and region was used to ensure the representativeness of the Polish population. All participants provided voluntary consent to participate in the study in the form of a written informed consent. The study was conducted in accordance with the Helsinki Declaration [40].

### 2.2. Outcome Variables

Behaviors concerning food purchases in the COVID-19 pandemic were addressed by two questions: “Have you bought more food than usual in the last month?” (answers: yes/no) and “What products do you buy in larger quantities than before?” (answers: cheese, processed cheese, and blue cheese, meat and cold cuts; bread; fruit and vegetables; bottled water; flour and sugar; canned meat and fish; pasta, groats, and rice; and concentrates and frozen foods). Respondents were also asked about their fears regarding the availability of food, namely “Are you afraid that due to the current situation related to the spread of the coronavirus, there may be serious restrictions in access to food?” Opinions were scored on a 5-point scale: 1—definitely not; 2—rather not; 3—neither no nor yes; 4—rather yes; 5—definitely yes.

### 2.3. Explanatory Variables

The explanatory variables included opinions on changes in food availability during the COVID-19 pandemic, trust in institutions and people as sources of information regarding current situation, perceived stress, and demographics such as gender, age, and education.

Perceived changes in food availability were assessed by the following question: “Have you noticed changes in the availability of food in stores over the last month?” The respondent could give the following answers: no, I did not notice any changes or yes. For the second answer, the respondent indicated the nature of the perceived changes: the amount of food available on the market has slightly decreased; the amount of food available on the market has significantly decreased; or there are shortages of some food products in stores.

The question “To which extent do you trust the following people or institutions as sources of information on the current situation?” was used to assess the trust in institutions and people as a source of information on the current situation during the pandemic. Definition of trust was not given in the questionnaire. Thus, the participants, when answering the question, used a subjective understanding of this concept. The respondents determined their trust toward the following entities: the World Health Organization (WHO), the European Union Institutions, the Ministry of Health, other government agencies, scientists, doctors, producers of food, mass media, bloggers, and friends or relatives. Opinions were scored on a 5-point scale: 1—I do not trust it/them at all; 2—I rather do not trust it/them; 3—I neither trust nor distrust it/them; 4—I rather trust it/them; 5—I definitely trust it/them.

Perceived Stress Scale 4 (PSS-4) was used to assess individuals’ perceived stress [41]. Participants were asked to indicate how often they felt or thought in a certain way: “In the last month, how often have you felt that you were unable to control the important things in your life?”; “In the last month, how often have you felt difficulties that were piling up so high that you could not overcome them?”; “In the last month, how often have you felt confident about your ability to handle your personal problems?”; and “In the last month, how often have you felt that things were going your way?”. Opinions of the respondents were scored on a 5-point scale: never, almost never, sometimes, fairly often, and very often. The answers to questions 1 and 2 were coded as follows: 0—never, 1—almost never, 2—sometimes, 3—fairly often, 4—very often, while the answers to questions 3 and 4 were coded inversely: 0—very often, 1—fairly often, 2—sometimes, 3—almost never, 4—never. Cronbach’s alpha coefficient for 4 items from this scale was 0.77, which implies that the scale can be considered reliable. The overall score of perceived stress was calculated as a sum of all ratings (range from 0 to 16 points). Higher scores correlated with higher perceived stress.

### 2.4. Statistical Analysis

Descriptive statistics, including frequency distributions and cross-tabulations, were performed. The normality of variables was assessed by the Kolmogorov–Smirnov test. The mean values and standard deviation (SD) were calculated.

Factor analysis was used to identify factors based on the scores on trust in all information sources. The factors were rotated by an orthogonal (Varimax) transformation. The number of factors was determined on the basis of the following criteria: components with an eigenvalue of 1, a scree plot test, and the interpretability of the factors. Information sources with factor loadings of at least 0.50 were considered. The factorability of the data was confirmed with the Kaiser–Meyer–Olkin (KMO) measure of sampling adequacy and Bartlett’s test of sphericity. The KMO value was found to be 0.775, and Bartlett’s test was significant at *p* < 0.0001 [42]. The following three factors were identified: “External institutions, scientists and physicians”, “Polish government institutions”, and “Mass media and friends”. The total variance explained was 62.9%. The explained variance for the three factors was 37.4%, 14.0%, and 11.4%, respectively. The factor-loading matrix is presented in Table 1.

To estimate the likelihood of occurrence of fear caused by anticipated limited access to food due to the spread of coronavirus, a logistic regression model (model 1) was developed. The dependent variable was the fear of limited access to food, which was treated as the dichotomous variable (yes versus no). The independent variables entered into the model were age, perceived stress, and identified factors related to trust (i.e., “External institutions, scientists and physicians”, “Polish government institutions”, and “Mass media and friends”) as continuous variables and perceived changes in food availability (dichotomous variable). To predict the purchase of a larger amount of food, another logistic regression model (model 2) was developed. The dependent variable was the purchase of a larger amount of food than usual in the month previous to the survey, which was treated as the dichotomous variable (yes versus no). The independent variables were the same as those used in the previous model plus the variable “fears of limited access to food”, which was also entered into the model. Correlation coefficient and cross-tabulation using the chi-square test were employed during the variable selection. Correlations between the independent variables in the correlation matrix were low (range: 0.001–0.124), except for the correlation between factors expressing trust in institutions and people as a source of information in the current situation (range: 0.315–0.411). Multicollinearity was examined through the variance inflation factor (VIF) and tolerance. The minimum observed value of tolerance was 0.886, which confirmed the lack of collinearity. The range of VIFs was 1.022–1.273, which did not confirm multicollinearity [43]. The variables were included in both models using the enter method. Nagelkerke’s R^2^ was used to assess the quality of the models [44]. The values of Nagelkerke’s R^2^ were 0.134 and 0.136 for models 1 and 2, respectively. Hypothesis H0 that all the parameters in the model are equal to 0 for both models was rejected at every level of significance, both for the likelihood ratio and the Wald statistics. The Hosmer–Lemeshow test was used to calculate the probabilities of all the observations divided into 10 groups. Odds ratios (ORs) and 95% confidence intervals (95% CI) for ORs were calculated. All analyses were performed using IBM SPSS Statistics for Windows, version 26.0 (IBM Corp., Armonk, NY, USA).

## 3. Results

### 3.1. Study Sample

The study sample consisted of 1033 participants (519 women and 514 men) aged between 18 and 65 years. Table 2 shows characteristics of the study sample. None of the variables differed from the value for the country as a whole.

### 3.2. Consumers’ Trust in Information Sources

Study participants showed the highest trust in sources such as physicians, scientists, and Polish Ministry of Health with regard to information about the situation during the COVID-19 pandemic. The lowest trust was recorded for bloggers, mass media, and government institutions other than the Ministry of Health. “External institutions, scientists, and physicians” was considered the most trusted source of information regarding the situation related to the COVID-19 pandemic, while “Mass media and friends” was the least trusted source of information (Table 3).

### 3.3. Perceived Stress

The sum of the scores representing the perceived stress ranged from 0 to 16 points, and the mean score was 6.8 ± 3.03. Extreme results were achieved for 25 people: 19 respondents obtained 0 points (lack of stress) and 6 respondents obtained 16 points (very high level of stress).

### 3.4. Perception of Food Availability during the COVID-19 Pandemic

Only 12.6% of respondents did not notice any changes in food availability in the month preceding the survey. More than 70% of respondents observed a decrease in the amount of food available on the market, while shortages of some foods were noticed by 16.2% of respondents (Table 4). The largest group of respondents did not anticipate restrictions in access to food due to the spread of the pandemic (definitely not and rather not: 43.7%), while the opposite opinion was expressed by 39.0% of the surveyed sample (Table 4). The mean value was 3.0, with a standard deviation of 1.13.

### 3.5. Purchase of Food in Larger Quantities

It was found that 44.0% respondents declared that they purchased larger amounts of food than usual in the month previous to the survey. They bought larger quantities of pasta, groats, and rice (33.0% of total sample); flour and sugar (27.9%); bottled water (20.7%); and meat and cold meats (20.4%). In the group of people who declared larger purchases, the proportion of people buying these products was as follows: 75.0%, 63.4%, 47.0%, and 46.4%, respectively. The smallest number of respondents indicated the purchase of larger amounts of fruits and vegetables, frozen foods, cheese, melted cheese, and blue cheese (Table 5).

### 3.6. Predictors of Consumers’ Fears and Purchase Behaviors during the COVID-19 Pandemic

Experiencing fears of limited access to food as the pandemic spreads (model 1) was more likely among people who felt more stress, those who had greater trust in sources of information on current situation, such as mass media, bloggers, and friends and relatives (“Mass media and friends”), and those who perceived changes in food availability in the month preceding the survey (Table 6). Each subsequent point indicating perceived stress increased the likelihood of experiencing fear by 16% (OR: 1.16; 95% CI: 1.11–1.22). Each subsequent point indicating trust in “Mass media and friends” increased the likelihood of experiencing fear by 50% (OR: 1.50; 95% CI: 1.22–1.85). However, the perceived changes in food availability were the strongest predictor of experiencing fears of limited access to food. Perception of such changes increased the likelihood of the occurrence of fears by more than three times (OR: 3.19; 95% CI: 2.03–5.01). In contrast, experiencing fears of limited access to food as the pandemic spreads was less likely among people who had greater trust in “Polish government institutions” (OR: 0.78; 95% CI: 0.66–0.91) (Table 6).

Similar to model 1, the purchase of larger quantities of food than usual in the month preceding the study was more likely among people with higher level of stress; those who had higher trust in sources of information on the current situation during the pandemic, such as mass media, bloggers, and friends and relatives (“Mass media and friends”); and those who perceived changes in food availability in the previous month (Table 6). Each subsequent point indicating perceived stress increased the likelihood of experiencing fear by 9% (OR: 1.09; 95% CI: 1.04–1.14). Each subsequent point indicating trust in “Mass media and friends” increased the likelihood of experiencing fear by 46% (OR: 1.46; 95% CI: 1.19–1.81). Perception of changes in food availability in the previous month increased the likelihood of purchasing larger quantities of food than usual in the previous month by 81% (OR: 1.81; 95% CI: 1.19–2.77). The strongest predictor of purchasing larger quantities of food than usual in the month preceding the survey was experiencing fears of limited access to food as the pandemic spreads, which increased the likelihood of such behavior by 130% (OR: 2.30; 95% CI: 1.75–3.03) (Table 6).

## 4. Discussion

Shortages or reduced food supplies in grocery stores were noted by the study participants (35.7% and 51.7%, respectively) and were also confirmed in other studies [45,46], which may be attributed to a change in existing purchasing behavior. However, only 44% of the respondents reported about major food purchases, which may indicate that other people did not feel threatened by the potential inability to meet their basic needs [47]. On the other hand, perceived changes in the food supply could be treated by other consumers as temporary, which was in line with the explanations offered by government institutions [15,45,46]. The obtained results showed that the response of Polish consumers on the food market in the initial phase of the pandemic was similar to that noted in other countries, i.e., a stock-up mentality began to appear [48,49]. Although supermarkets in most countries have introduced changes to reduce panic buying and to minimize the spread of COVID-19 while shopping, the panic-stricken behaviors were not completely eliminated [50]. Thus, unless policy makers can find a way to restore a sense of security, the cycle of panic buying, hoarding, and scarcity may continue to exist [37].

Findings of the present study confirmed the relationship between perceived stress and both fears of limited access to food and purchases of larger quantities of food than usual [51]. Perceived stress was a stronger predictor of the fears than of the purchase behaviors. In previous pre-pandemic studies, different mean values for stress perception were reported when PSS-4 scale was used, for example, the mean values were 6.11 in a British sample [52], 5.43 in a Spanish sample [53], and 6.27 in a Korean sample [54]. The mean value of perceived stress in our sample (6.83) was higher than that reported in the abovementioned studies. This can be explained by the study period, i.e., the beginning of the pandemic, which was associated with many ambiguities as well as terrible information about the situation in other countries that were more affected by the coronavirus at that time (China and Italy). It could have been expected that the situation in Poland and around the world related to the pandemic during the study would cause much higher stress. However, the measurement tool (PSS-4), which examines coping in general [53], without referring directly to a pandemic, could have had a decisive impact on the obtained results. Thus, implementing other tools to measure the level of stress during pandemic is also recommended. Despite this limitation, the obtained results showing an increasing likelihood of occurrence of both fear associated with limited food availability and the purchase of larger than usual quantities of food with the increase in perceived stress, require the latter to be focused upon to limit the negative effects of a pandemic.

Experiencing fear during a pandemic, which is caused amongst others by changes in food availability, raises the question of the importance of available information and trust in their sources in reducing this negative emotion and its consequences, e.g., panic-stricken purchasing behavior. Trust as an element of social capital [55] has been found to be important in risk communication and management [25,56], in interactions among people, and in civic engagement [57]. Thus, trust gains importance during a pandemic because it facilitates the transformation of available passive information into information that is usable in decision-making [58]. Moreover, according to previous studies, trust plays an important role in influencing attitudes and purchasing decisions regarding food products [25,59,60]. In the present study, researchers’ attention concerning trust was focused solely on relationships between individuals (interpersonal or relational) and between individuals and institutions as sources of information on the pandemic situation. However, trust was measured only at the general level, without recalling different spheres of the pandemic situation. External institutions represented by WHO and EU institutions and experts who are most involved in combating the pandemic, i.e., physicians and scientists, were recognized as the most trusted sources of information (“External institutions, scientists, and physicians”—mean 3.6 ± 0.73). Public trust in science has remained stable for almost 50 years [60,61] and is still strong. However, the perception of the “experts” that is also continuously changing during the pandemic may cause a decline in trust [62]. An erosion of public trust in expertise in general, rather than in science, is currently observed [63]. The traditional roles of “information gathering” experts are diminished by the accelerating tempo of dissemination of information regardless of quality, which is constantly being observed in mass media. This implies that the recipient of the message has a problem in differentiating between valuable and worthless information, which further reduces public trust in expertise. Moreover, the media tend to sensationalize the reality or present contradictory information, therefore creating distrust in them, which often results from the dramatization of the received information [64]. In the present study, trust in “Mass media and friends” was the lowest compared to that in other sources of information, which may confirm the above discussed limitations of this source of information and its public perception. However, mass media together with bloggers, friends, and relatives were the predictors of both fears of limited access to food and purchase of larger than usual quantities of food as the pandemic spreads. Greater trust in these sources of information increased the likelihood of participants’ fear of food shortages and their food-gathering behaviors.

Conversely, greater trust in national government institutions reduced the likelihood of participants’ fears of food shortages, but this trust was not a predictor of behavior associated with the purchase of larger amounts of food. The positive effect of trust in government institutions was also observed in other studies [65,66]. In our study, the trust in Polish government was relatively low (mean 3.2 ± 0.97). However, the trust in the Polish Ministry of Health (3.7 ± 1.08) was higher than that in other government agencies (3.0 ± 1.04) and European institutions (3.2 ± 1.01), and at the same time, it was almost equal to trust in WHO (3.66 ± 0.95). High trust in WHO may be linked to its daily reports assessing not only the overall extent of the crisis but also its progression [67]. In the case of the COVID-19 pandemic, however, many individuals raised their voices to undermine the credibility of health institutions, which can be difficult to rebuild especially during a pandemic [68]. Thus, monitoring the level of public trust as the pandemic progresses is recommended in future studies in order to counteract the decline in trust in health institutions, which are of great importance in the pandemic.

According to the Trust and Confidence Model, trust can indirectly influence the acceptance of recommendations [35,36]. Nevertheless, it is noted that the positive effect of trust does not imply that all recommendations are followed. For example, trust in the government positively influenced an intention to accept vaccination, but not to an intention to adopt protective measures (such as adopting additional hygienic precautions) [65], which can significantly limit the effect of governmental measures to control the spread of pandemic by imposing many restrictions. In addition, the effect of trust in government institutions may change as the pandemic evolves. For example, during the influenza A (H1N1) pandemic, a significant reduction in government trust was noted as the pandemic continued [38,65]. Decreased trust in the government’s ability to handle the threat may result from conflicting messages that can create skepticism about public health warnings [31,36]. People believed that at the beginning of the pandemic, information was hidden or kept secret from them, while most believed that the government later exaggerated the situation [69,70]. Thus, responsible communication regarding the crisis is required to maintain and build trust in the government during a pandemic [35].

Although the results of the present study refer to the beginning of the pandemic, they have shown how much food purchasing behaviors in uncertain times depend on the level of perceived stress and trust in various sources of information. A highly dynamic situation in the pandemic, in terms of illnesses and the level of health care, food market situation, people’s mental health, and relation to trust in information sources, requires continuous monitoring.

The present study provides an insight into how a state of epidemic combined with lockdown can affect food purchase behaviors; however, there are some limitations that need to be considered. First, the study used an approach that provides a general summary of the changes in food buying behaviors in the very early stage of a pandemic, i.e., purchase of larger amounts of food and its determinants. Thus, the results cannot be interpreted in the context of long-term effects. This suggests the need for cautious data interpretation and calls for further studies in this field. The measurement tool (PSS-4) concerns coping in general [53], without referring directly to a pandemic, which is also a limitation of the present study. Application of other tools to measure the level of stress during pandemic is recommended in further research. Lastly, this was a cross-sectional study that did not allow for an assessment of the causality of relationships between the variables. Our findings are specific to the Polish population at a certain stage of the COVID-19 pandemic and should not be generalized to populations of other cultural backgrounds. However, the observations could be of potential use beyond Poland, not only in designing further research that allows to observe changes during the pandemic but also in developing strategies to improve the effectiveness of communication in pandemic conditions, taking into account the public trust.

The strength of our study is the fact that the survey was conducted in the beginning of the COVID-19 pandemic in Poland, just as the lockdown was being introduced. Therefore, the bias of self-reported food purchase behaviors was not influenced by experiences from the further period of the pandemic.

## 5. Conclusions

The present study revealed less trust in media than in other sources of information on the COVID-19 pandemic in Poland. Moreover, trust in information from media and other people (friends and bloggers) increased the likelihood of fears of limited access to food as the pandemic continued to spread and increased the likelihood of purchasing larger quantities of food than usual. Therefore, government institutions may have difficulty in disseminating pandemic-related recommendations through media organizations, due to relatively low trust in them and their role in not only increasing the likelihood of occurrence of fears regarding food availability but also in causing panic-stricken food buying behaviors. Thus, it is necessary for government agencies, which consider the opinions of physicians and scientists when formulating their policies, to communicate with the public through more direct channels, including government-operated websites, television, and radio networks. Thus, it may also be possible to reduce the predicted effect of the “Mass media and friends” of increasing the likelihood of occurrence of food availability fears and of food purchase behaviors related to uncertainty in the pandemic. In the first stage of the COVID-19 pandemic, the likelihood of occurrence of both fears regarding food availability as the pandemic spreads and the purchase of larger quantities of food than usual increased when higher perceived stress was claimed. Therefore, further studies are required to understand whether COVID-19-related lockdown has resulted in long-term changes both in the level of public trust toward information sources and in food purchase behaviors. The latter can promote long-term reinforcement of adverse eating habits and related health problems. Knowing how people function in the food sphere during a pandemic is also pertinent to understand the role that food may play in future epidemic quarantines.

## Figures and Tables

**Table 1:** Factor-loading matrix.

Variables	“External Institutions, Scientists and Physicians” (Factor 1)	“Polish Government Institutions” (Factor 2)	“Mass Media and Friends” (Factor 3)
World Health Organization (WHO)	0.806 *	0.194	0.041
EU institutions	0.723 *	0.031	0.220
Polish Ministry of Health	0.290	0.829 *	0.015
Other government agencies	0.112	0.895 *	0.126
Scientists	0.733 *	0.191	0.054
Physicians	0.716 *	0.215	0.076
Food producers	0.303	0.473	0.422
Mass media	0.185	0.447	0.571 *
Bloggers	−0.052	0.177	0.794 *
Friends and relatives	0.188	−0.095	0.727 *

* Factor loadings ≥ 0.5.

**Table 2:** Study sample characteristics.

Variables		*N* = 1033 *	%
Gender	Female	519	50.2
	Male	514	49.8
Education	Lower than upper secondary	393	38.0
	Upper secondary	382	37.0
	Higher	258	25.0
Place of residence	Rural area	395	38.2
	City ≤ 200,000 residents	419	40.6
	City > 200,000 residents	219	21.2
Age	18–24 years old	155	15.0
	25–34 years old	227	22.0
	35–44 years old	238	23.0
	45–54 years old	227	22.0
	55–65 years old	186	18.0
Age in years (mean ± standard deviation)		39.9 ± 13.1	

* *N*—number of participants.

**Table 3:** Consumers’ trust in selected sources of information on the COVID-19 pandemic.

Factor	Variables	Mean * ± Standard Deviation	Factor (Mean ** ± Standard Deviation)
“External institutions, scientists, and physicians”	World Health Organization (WHO)	3.6 ± 0.95	3.6 ± 0.73
	EU institutions	3.2 ± 1.01	
	Scientists	3.7 ± 0.90	
	Physicians	3.9 ± 0.91	
“Polish government institutions”	Polish Ministry of Health	3.7 ± 1.08	3.2 ± 0.97
	Other government agencies	3.0 ± 1.04	
“Mass media and friends”	Mass media	2.8 ± 1.02	3.0 ± 0.71
	Bloggers	2.7 ± 0.98	
	Friends and relatives	3.4 ± 0.85	
	Food producers	3.1 ± 0.93	

* Five-point scale: 1—I do not trust it/them at all; 2—I do not really trust it/them; 3—I neither trust nor distrust it/them; 4—I rather trust it/them; 5—I definitely trust it/them. ** Calculated after summing the scores for individual items within a given factor and dividing by the number of items in it (range 1–5).

**Table 4:** Opinions on availability of food during the COVID-19 pandemic.

Variable		*N* *	%
Perceived changes in food availability in the previous month	No, I did not notice any changes	131	12.6
	Yes, the amount of food available on the market has slightly decreased	367	35.5
	Yes, the amount of food available on the market has significantly decreased	167	16.2
	Yes, there are shortages of some food products in stores	369	35.7
Fears of limited access to food as the pandemic spreads	Definitely not	58	5.6
	Rather not	394	38.1
	Neither no nor yes	179	17.3
	Rather yes	308	29.8
	Definetely yes	95	9.2

* *N*—number of participants.

**Table 5:** Foods purchased in larger quantities during the COVID-19 pandemic.

Food Groups	Total Sample (*N* = 1033 *)		Group Declaring Purchasing Larger Quantities of Food than Usual in the Previous Month (*N* = 454 *)			
			Product Purchased in Larger Quantities than Usual		Product not Purchased in Larger Quantities than Usual	
	*N* *	%	*N*	%	*N*	%
Pasta, groats, and rice	340	33.0	340	75.0	114	25.0
Flour and sugar	288	27.9	288	63.4	166	36.6
Bottled water	213	20.7	213	47.0	241	53.0
Meat and cold meats	211	20.4	211	46.4	243	53.6
Canned meat and fish	188	18.2	188	41.4	266	58.6
Bread	145	14.1	145	32.0	309	68.0
Concentrates	146	14.0	146	32.2	308	67.8
Cheese, blue cheese, and melted cheese	124	12.0	124	27.3	330	72.7
Frozen foods	124	12.0	124	27.4	330	72.6
Fruits and vegetables	83	8.0	83	18.2	371	81.8

* *N*—number of participants.

**Table 6:** Odds ratios (OR; 95% CI) of fears and purchase behaviors of the study sample.

Variables	Fears of Limited Access to Food as the Pandemic Spreads (Model 1)		Purchase of Larger than Usual Quantities of Food in the Previous Month (Model 2)	
	No	Yes	No	Yes
Age (in years)	1	0.99 (0.98–1.00)	1	1.00 (0.99–1.021)
Perceived stress (in points)	1	1.16 *** (1.11–1.22)	1	1.09 *** (1.04–1.14)
“External institutions, scientists, and physicians”	1	0.93 (0.76–1.15)	1	1.12 (0.91–1.38)
“Polish government institutions”	1	0.78 ** (0.66–0.91)	1	0.91 (0.77–1.06)
“Mass media and friends”	1	1.50 *** (1.22–1.85)	1	1.46 *** (1.19–1.81)
Perceived changes in food availability in the previous month	1	3.19 *** (2.03–5.01)	1	1.81 ** (1.19–2.77)
Fears of limited access to food	-	-	1	2.30 *** (1.75–3.03)

** *p* < 0.01; *** *p* < 0.001; (Wald’s test).
